# Bile Cell-Free DNA as a Liquid Biopsy for Biliary Tract Malignancies: A Scoping Review

**DOI:** 10.3390/cancers18203251

**Published:** 2026-10-09

**Authors:** Arjun Dhir, Brian Martins, Zachary P. Yeung, Neil R. Sharma, Mohit Girotra

**Affiliations:** 1 Department of Internal Medicine, University of Arizona College of Medicine—Phoenix, Phoenix, AZ 85004, USA; 2 Department of Medicine, University of Arizona College of Medicine—Phoenix, 475 N. 5th Street, Phoenix, AZ 85004, USA; brianmartins@arizona.edu; 3 Banner MD Anderson Cancer Center, Banner Health, Phoenix, AZ 85234, USA; zachary.yeung@bannerhealth.com (Z.P.Y.); neil.sharma@bannerhealth.com (N.R.S.); 4 Digestive Health Service Line, Banner Health, Phoenix, AZ 85006, USA; mohit.girotra@bannerhealth.com

**Keywords:** bile, cell-free DNA, cholangiocarcinoma, liquid biopsy, biliary tract cancer, next-generation sequencing, molecular profiling

## Abstract

**Background:** Biliary tract cancers (BTCs) carry a poor prognosis driven by frequent diagnosis at advanced or metastatic stages. Inadequate tissue sampling impedes biomarker-directed selection of therapy that could potentially improve outcomes over limited chemotherapeutic options. Plasma cell-free DNA (cfDNA) yields limited sensitivity. However, given its direct contact with the tumor, bile holds promise as a locally enriched source of tumor-derived cfDNA for evaluation. **Methods:** Following the PRISMA-ScR guidelines, we searched PubMed/MEDLINE and EMBASE without date restrictions. Seventeen primary studies of bile cfDNA in cholangiocarcinoma (CCA), gallbladder carcinoma (GBC), pancreatic ductal adenocarcinoma (PDAC), and primary sclerosing cholangitis (PSC) were synthesized narratively. **Results:** Compared to plasma cfDNA, Bile cfDNA was isolated at higher concentrations and in longer fragments than plasma cfDNA; bile cfDNA demonstrated higher concordance with matched tumor tissue, capturing more actionable alterations. Pairing it with serum CA19-9 or newer bile-derived markers improved performance. Emerging data suggest a role in early detection of CCA during PSC surveillance. **Conclusions:** Bile cfDNA as a liquid biopsy medium offers analytic advantages over plasma for detecting and profiling biliary malignancy. Standardized collection, extraction, and reporting protocols and prospective multicenter validation are needed before routine use.

## Simple Summary

Cancers of the bile ducts and gallbladder are usually discovered late, when treatment options are limited. Doctors normally look for these cancers by brushing cells from the narrowed duct during an endoscopic procedure, but this approach misses more than half of them. Blood tests that search for tumor genetic material also perform poorly, because these tumors shed very little of it into the bloodstream. Bile, the fluid that washes directly over the tumor, can be collected during the same endoscopic procedure without added risk. This review brings together seventeen studies that examined tumor genetic material in bile. Across these studies, bile contained far more tumor genetic material than blood, matched the tumor itself more closely, and revealed changes that could guide treatment. Agreed methods for collecting and processing bile are still needed before this approach can be used routinely.

## 1. Introduction

Biliary tract cancers (BTCs), comprising intrahepatic (iCCA), perihilar (pCCA), and distal cholangiocarcinoma (dCCA), along with gallbladder carcinoma (GBC), are aggressive tumors that usually present as advanced unresectable or metastatic disease with five-year relative survival rates near 10% in advanced disease [1]. Surgical resection is the only curative option, yet fewer than 30% of patients undergo resection at diagnosis due to nonspecific presenting symptoms, such as jaundice, abdominal pain, and weight loss, and early-detection tools are limited.

Workup of a biliary stricture incorporates cross-sectional imaging, endoscopic retrograde cholangiopancreatography (ERCP) with brush cytology, and serum carbohydrate antigen 19-9 (CA19-9) evaluation. Each of these components has analytical limitations: brush cytology confers a meager 45% sensitivity for malignancy [2], and CA19-9 specificity is confounded by benign obstruction, cholangitis, or primary sclerosing cholangitis (PSC). The result is diagnostic uncertainty and delay, most consequential in PSC, where the lifetime risk of CCA reaches 10–15% and surveillance remains inadequate [3].

Table 1 summarizes the diagnostic performance of the modalities currently used in this setting.

Liquid biopsy offers a minimally invasive alternative. Plasma cell-free DNA (cfDNA) has emerged as a promising molecular marker for cancer detection readily accessible by phlebotomy. However, in BTC it falls short when used in isolation. Circulating tumor DNA (ctDNA) is detected in only 10–50% of cases even on sensitive next-generation sequencing (NGS) platforms [7,8,9,10,11,12,13], likely because biliary tumors shed little DNA into the circulation. Bile offers a different medium for liquid biopsy, as sampling of bile during ERCP or percutaneous transhepatic cholangiodrainage (PTCD) offers genetic material shed directly from the malignant biliary epithelium. This liquid biopsy modality samples a locally concentrated cfDNA reservoir in contact with the tumor [14]. While bile presents a rich source of tumor DNA, the lack of standardized clinical protocols has stalled its adoption. Thus, this review synthesizes 17 primary studies spanning bile cfDNA biology, analytical considerations, diagnostic performance, and emerging applications. By critically evaluating these data, the objective of this review is to examine bile cfDNA in bridging the current diagnostic gap and better characterizing critical questions regarding its clinical sensitivity, molecular concordance with tissue, and immediate utility in guiding genotype-directed oncological care.

**Figure 1:**
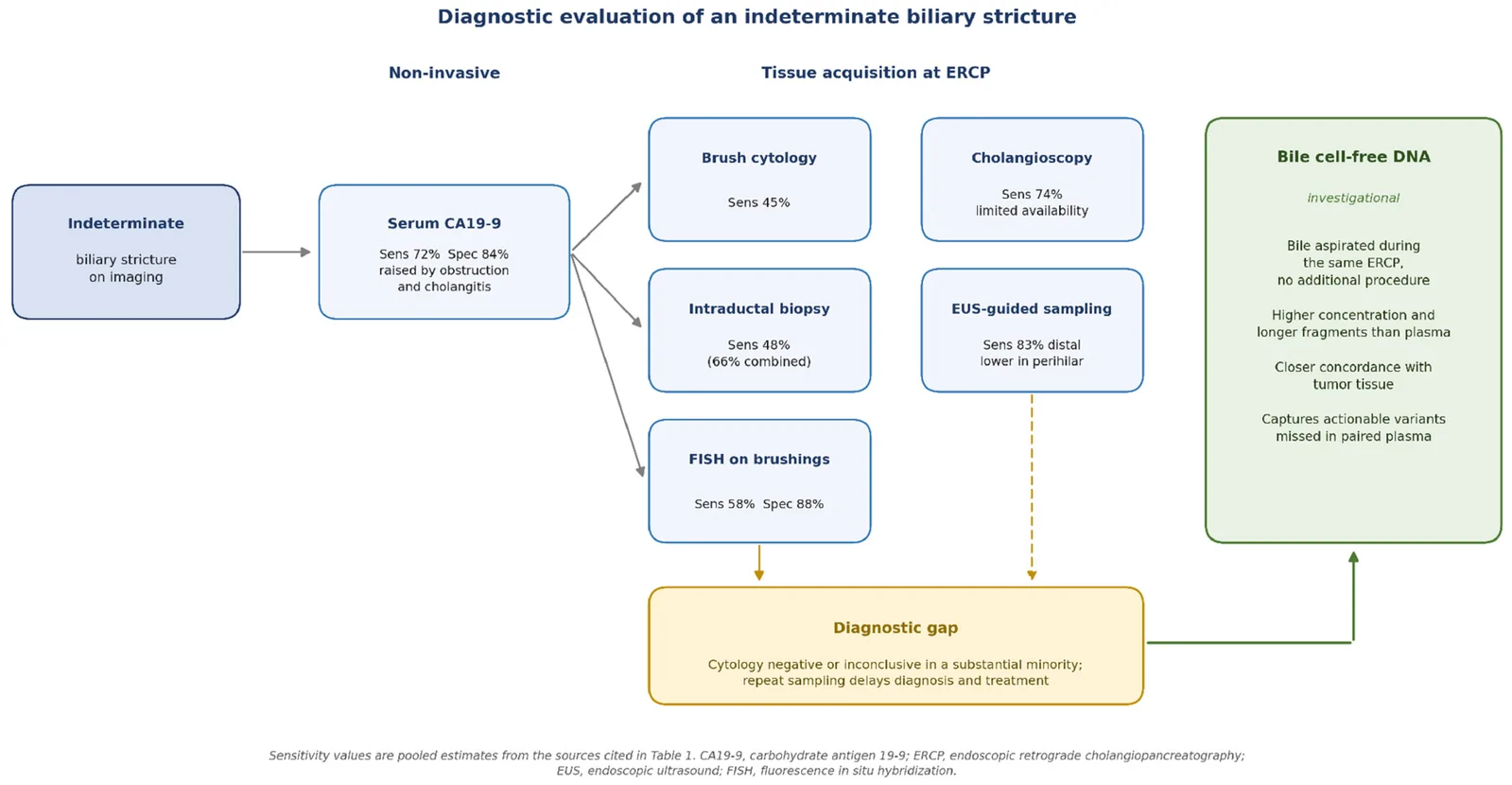
Diagnostic evaluation of an indeterminate biliary stricture and the position of bile cell-free DNA analysis. Sensitivity values are the pooled estimates summarized in Table 1. Bile is aspirated during an endoscopic retrograde cholangiopancreatography that is already indicated, and therefore adds no incremental procedural risk. All bile cell-free DNA applications shown remain investigational.

Figure 1 situates bile cell-free DNA analysis within this diagnostic pathway.

## 2. Methods

This scoping review followed the framework of Arksey and O’Malley [15] and is reported in accordance with the PRISMA-ScR statement [16].

### 2.1. Search Strategy and Eligibility

PubMed/MEDLINE and Embase were both searched on 14 September 2026 with no date or language restrictions, combining terms such as “bile AND cell-free DNA,” “bile AND liquid biopsy,” “biliary AND cfDNA,” and “cholangiocarcinoma AND circulating tumor DNA.” Studies qualified if they enrolled patients with biliary pathology (CCA, GBC, PDAC with biliary involvement, or PSC), analyzed cfDNA from bile, and reported original data on methodology, platform validation, diagnostic performance, molecular profiling, or outcomes. Animal and in vitro work, plasma- or serum-only analyses, abstracts without full-text data, and narrative reviews were excluded. Plasma cfDNA search terms were included deliberately so that plasma-based studies could be identified as the comparator arm and excluded at screening. The full search strategies and line-by-line yields for both databases are provided in Supplementary Tables S1–S3. This review was conducted and reported in accordance with the PRISMA 2020 statement and the PRISMA extension for Scoping Reviews (PRISMA-ScR) [16]. No review protocol was prepared or registered.

### 2.2. Study Selection and Synthesis

**Figure 2:**
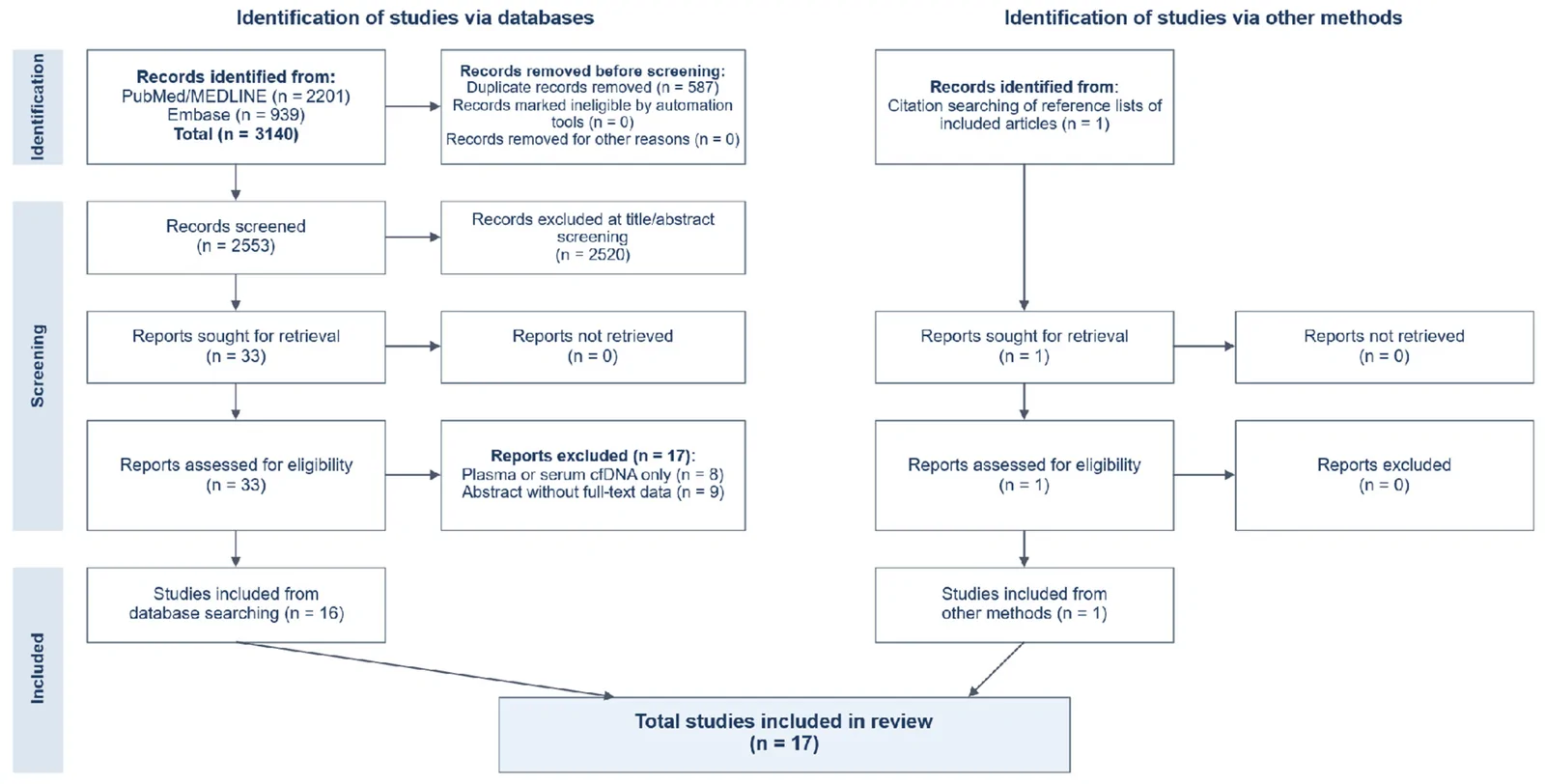
Identification and selection of studies. PubMed/MEDLINE and Embase were searched on 14 September 2026 with no date or language restrictions; duplicates were removed manually in Zotero. Full search strategies and line-by-line yields are given in Supplementary Tables S1 and S2. Adapted from the PRISMA 2020 flow diagram.

Titles and abstracts were screened independently by two reviewers (A.D., B.M.), with disagreements resolved by discussion, followed by full-text assessment; reference lists of included papers were hand-searched. Records were deduplicated manually in Zotero, and non-English articles were excluded at the screening stage. The selection process is summarized in Figure 2, and the characteristics of included studies are in Table 2. Given heterogeneity in design, cohort size (10–96 patients), platform (28–520-gene panels, ddPCR, WGS), and reporting, quantitative meta-analysis was not feasible. Data were charted on a standardized form capturing author, year, journal, sample size, cancer type, collection method, extraction protocol, platform, diagnostic metrics, mutational spectrum and actionable alterations, and outcomes.

### 2.3. Methodological Limitations of the Review

Consistent with scoping review methodology, formal risk-of-bias tools (e.g., QUADAS-2, Newcastle Ottawa Scale) were not applied, since the aim was to map the evidence rather than pool estimates. Gray literature, preprints, and non-English publications were not systematically captured, and the small number of eligible studies, mostly single-center and retrospective, further limits generalizability.

## 3. Biological Rationale for Bile Cell-Free DNA

### 3.1. Fragment Size Distribution

A key feature of bile cfDNA is its fragment size distribution, which is significantly larger than that of plasma cfDNA (~147–167 bp). Gel electrophoresis of bile from BTC patients demonstrates predominantly long-fragment DNA in the kilobase range [17], and analyses of pancreaticobiliary stricture specimens reveal high-molecular-weight fragments of 8000–10,000 bp [19]. This size difference likely reflects reduced nuclease-mediated degradation of DNA within the biliary compartment compared with plasma. The clinical implication is that extraction kits designed for short plasma cfDNA may underperform on bile samples, requiring protocols capable of recovering longer DNA species [18].

### 3.2. Concentration Enrichment Relative to Plasma

Bile cfDNA concentrations consistently exceed those of plasma across studies analyzing diverse collection methods and patient populations. Reported mean concentrations have reached 1918 ng/mL in bile versus 63.1 ng/mL in paired plasma samples (*p* = 0.0017) [12]. Additionally, in patients with CCA that can be resected, bile cfDNA concentrations measured 1433.3 versus 17.1 ng/mL in plasma (*p* < 0.001) [8], and 886.10 ± 182.3 in bile versus 40.52 ± 7.46 ng/mL in plasma in 68 stricture patients undergoing ERCP [20]. The enrichment of cfDNA in bile is highlighted by an independent cohort study, which demonstrated an estimated 68.2-fold increase in bile cfDNA concentration compared to plasma [9]. The consistent enrichment of bile cfDNA across diverse cohorts and its superior performance as a liquid biopsy source may be explained by the direct shedding of tumor DNA into bile, thereby bypassing the dilution that weakens ctDNA signals in plasma.

## 4. Pre-Analytical and Analytical Considerations

### 4.1. Collection Methods

Bile is obtained in three main ways: ERCP-guided aspiration (most common), PTC, and intraoperative sampling. When comparing PTC and intraoperative specimens, there appeared to be no meaningful difference in fragment size or single-nucleotide variant (SNV) detection [17], suggesting the collection method does not substantially alter bile cfDNA composition. Serial sampling is practical: 212 specimens were obtained from 87 patients during drainage procedures, supporting repeat testing in routine workflows [10].

### 4.2. cfDNA Extraction and Specimen Stability

As noted in Section 3.1, the predominance of long-fragment DNA in bile may cause standard plasma cfDNA extraction kits to underperform. However, a bile-specific extraction method (“3D-BCF”) captures fragments spanning 40 bp to 10 kb and has demonstrated superior yield over four commercial kits designed for shorter plasma fragments (~167–200 bp) [18]. Optimizing extraction protocols and technologies to capture the true fragment profile of bile cfDNA is necessary to generate maximum yield to achieve accurate mutation detection in biliary malignancies. In addition to selecting the right extraction protocols, maintaining the integrity of the sample through storage and processing is essential. Available evidence demonstrates that bile collected in tubes containing a nucleic-acid protectant (BEAVER cfDNA tubes) remains stable for up to 10 days at room temperature, whereas protectant-free polypropylene tubes lose approximately 25% of recoverable cfDNA by day 7, with progressive loss of long fragments [21]. The same protectant preserves integrity for two months at 4 °C, while protectant-free samples require freezing at −20 °C or below [21]. Proteins and ctDNA in drainage bile remain stable at room temperature for approximately 7 h [24]. Notably, standard blood collection tubes (such as K2EDTA and Streck BCT) remain unvalidated in bile, and stability data from plasma cannot simply be extrapolated to the complex alkaline bile fluid. Without tubes that preserve bile cfDNA at room temperature for several days, testing cannot scale to centralized reference labs and remains confined to centers with on-site processing. Nonetheless, the evidence summarized in Table 3 indicates that collecting bile directly into specialized preservative tubes can stabilize cfDNA during delayed transport, preserving sample integrity for downstream genomic analysis.

### 4.3. Analytical Platforms

To capture the genomic profile of pancreatobiliary malignancies, targeted next-generation sequencing (NGS) panels have emerged as the most widely adopted analytical approach. A validated “Bilemut” workflow based on the Oncomine Pan-Cancer Cell-Free assay (52 genes) has been applied in both indeterminate stricture and PSC surveillance cohorts [20,25], while larger panels enable tumor mutation burden (TMB) estimation and structural variant detection directly from bile [8]. To minimize sequencing noise in larger panels, machine learning–based classifiers have been integrated to differentiate true somatic variants from artifacts, thereby enhancing variant calling accuracy [9]. While NGS panels offer breadth with their broad genomic profiling, droplet digital PCR (ddPCR) offers depth and sensitivity for targeting specific mutations, such as Kirsten rat sarcoma viral oncogene homolog (*KRAS*) [11]. This heightened sensitivity builds on foundational work using LigAmp technology, which demonstrated *KRAS* alteration in 87.5% of samples of neoplastic bile compared to 32.1% in benign control specimens [14]. Moving beyond single-gene loci, the field has increasingly adopted global genomic characterization via whole-genome sequencing (WGS) [22]. WGS offers a comprehensive view of the cholangiocarcinoma genome by detecting structural variants, copy number alterations, and complex rearrangements that remain undetected by targeted NGS panels. As of now, no bile cfDNA assay is commercially available yet; however, the numerous advantages of bile as a substrate for liquid biopsy may make it a reasonable target.

### 4.4. Bile Composition as a Pre-Analytical Variable

Bile is a chemically dynamic medium, and its composition was not treated as a pre-analytical variable in any study reviewed here. Biliary bile acid concentration, and the ratio of conjugated to unconjugated and of hydrophobic to hydrophilic species, vary substantially between individuals and within the same individual across the day under circadian and postprandial influence [26]. This variability is mechanistically relevant in two respects. Hydrophobic bile acids, principally deoxycholic and chenodeoxycholic acid, induce apoptosis in epithelial cells in proportion to their relative hydrophobicity [27], and deoxycholic acid generates reactive oxygen species sufficient to produce measurable DNA strand damage above a concentration threshold [28,29]. Bile rich in hydrophobic species might therefore be expected to contain a greater total quantity of cell-free DNA through increased apoptotic release from biliary epithelium, while that DNA may be more fragmented and of lower integrity. Hydrophilic species such as ursodeoxycholic acid are cytoprotective and would be predicted to exert the opposite effect. Whether these opposing influences materially alter tumor-derived fraction, fragment size distribution, or variant allele frequency in bile remains unknown, and no study has reported bile acid profiling alongside cell-free DNA quantification. Because fasting state, ursodeoxycholic acid therapy, and the degree of biliary obstruction each alter bile acid composition, and because all three are common in this population, standardization of sampling conditions and parallel bile acid measurement warrant inclusion in future protocols.

## 5. Diagnostic Performance

### 5.1. Concordance with Tumor Tissue

In matched-sample studies, bile cfDNA demonstrates concordance with tumor tissue more consistently than plasma cfDNA (Table 4), with bile-versus-plasma mutation detection of 96.2% versus 31.6% [7], 90% versus 35% [12], and 70% versus 10% [8] across distinct cohorts. Notably, tier-1 actionable variants such as *BRAF* V600E mutations and *FGFR2* fusions were identified in matched bile and tissue specimens but not in paired plasma [8], showing how bile as a source of cfDNA improves targeted-therapy actionability.

### 5.2. Comparison with Brush Cytology

In head-to-head comparisons in pancreaticobiliary strictures, bile cfDNA NGS was substantially more sensitive than ERCP brush cytology (53% vs. 19%; *p* = 0.016) [19]. Cytology can visually confirm intact tumor cells on the brush; however, bile cfDNA can flag molecular alterations when cytology is inconclusive. Although this evidence is derived from a single-center cohort of 50 patients, available data suggest that bile cfDNA and cytology may serve complementary roles within ERCP-based diagnostic workups.

### 5.3. Combined Biomarker Strategies

Combining bile cfDNA with conventional biomarkers demonstrates superior diagnostic yield. Combining bile cfDNA with serum CA19-9 achieved a sensitivity of 96.4% in one cohort, exceeding either biomarker alone (bile cfDNA 71.4%; plasma cfDNA 53.6%) [13]. Two bile-derived markers, Syncytin-1 and *SLC7A11*, identified in 48 CCA and 48 gallstone patients, achieved a three-marker AUC of 0.927 with CA19-9 (versus 0.857 for CA19-9 alone), with Syncytin-1 alone reaching an AUC of 0.805 and 87.0% specificity [23]. These findings indicate that integrating bile cfDNA with complementary biomarkers may improve diagnostic accuracy while sparing additional invasive sampling.

### 5.4. Strength of the Underlying Evidence

The performance estimates summarized above derive from small, predominantly single-center and retrospective cohorts and require corresponding caution. Enrollment in the included studies was restricted to patients already undergoing endoscopic retrograde cholangiopancreatography or percutaneous drainage for a clinically or radiologically suspected malignant stricture, which concentrates the spectrum of disease toward advanced or overt tumors. Where comparator groups were included, they comprised benign biliary disease or healthy liver donors rather than the diagnostically uncertain strictures in which such an assay would be deployed. No included study reported blinded adjudication against a prespecified reference standard. Concordance estimates derived from fewer than 15 paired specimens [8,12] are particularly susceptible to sampling variation. These features favor higher apparent sensitivity and tissue concordance than would be anticipated in unselected practice, and the estimates presented here are best regarded as evidence of analytic feasibility rather than of validated diagnostic performance. Section 8 addresses the resulting implementation challenges in further detail.

## 6. Molecular Profiling and Therapeutic Relevance

### 6.1. Mutational Spectrum and Actionable Alterations

The mutations recovered from bile cfDNA track the known molecular landscape of biliary tract cancers, though the mix of actionable alterations shifts with primary anatomic site and should be interpreted accordingly (Table 5).

#### 6.1.1. Pancreatic Ductal Adenocarcinoma (PDAC)

*KRAS* is the dominant driver of pancreatic ductal adenocarcinoma, present in more than 90% of tumors and already detectable in pancreatic intraepithelial neoplasia, the earliest recognized precursor lesion [30]. Mutations cluster largely at glycine 12, with G12D (42%), G12V (31%), and G12R (15%) predominating. G12C, the allele targeted by the first approved covalent inhibitors, accounts for fewer than 2% of cases [30,31]. Given this background, the yield of bile cfDNA in pancreatic strictures is modest: in a 40-patient bile cohort, pathogenic alterations were detected in only 2 of 5 bile samples (40%) from patients with PDAC, and *KRAS* specifically was identified in 7 of 32 (22%) bile samples across all pancreaticobiliary malignancies [19]. Finding *KRAS* in only 40% of cases, when more than 90% harbor the mutation, reveals a high false-negative rate. The authors attributed this limitation to a 2% mutant allele frequency reporting floor, which is substantially higher than the 0.1–0.15% thresholds used in modern bile cfDNA studies, filtering out low-abundance tumor DNA [19,20]. *TP53* tumor suppressor alterations appear in 47.4% of Bilemut-positive cases [20], where Bilemut-positivity denotes samples having at least one somatic mutation detected by the study’s targeted NGS bile cfDNA panel. Using WGS in cholangiocarcinoma specimens, *TP53* mutations were found in 27.3% of samples [22]. *TP53* and *KRAS* co-mutation is characteristic of PDAC and is described in resected tumor tissue [32]. The frequencies cited above, however, derive from bile in patients with biliary strictures [20], not from tumor tissue, and therefore reflect what is shed into bile rather than the underlying tumor genotype. *GNAS* mutations, typical of intraductal papillary mucinous neoplasms (IPMNs, a PDAC precursor), appeared in 15.8–30% of cases across cohorts [20,25], suggesting bile sampling and analysis could help stratify IPMN-associated malignant progression.

#### 6.1.2. Extrahepatic (eCCA) and Perihilar Cholangiocarcinoma

Perihilar and distal extrahepatic CCA are enriched for *KRAS*, *TP53*, and *SMAD4* relative to iCCA [33]. *KRAS* mutations are found in moderate frequency, whereas *TP53* remains relevant, reported in 47.4% of Bilemut-positive cases in a cohort of 68 patients with biliary strictures (70.6% distal, 25% perihilar locations) [20]. In this cohort, *ERBB2* amplifications (8.8%) and *ERBB3* mutations (22.8%) were identified in bile cfDNA [20]. Of these, only *ERBB2* is currently actionable with zanidatamab, which is a bispecific antibody that binds epitopes of the HER2 receptor encoded by *ERBB2*, and received accelerated FDA approval in November 2024 for previously treated, unresectable or metastatic HER2-positive biliary tract cancer as evidenced by the HERIZON-BTC-01 trial [34]. *ERBB3* alterations have no approved targeted therapy in biliary tract cancer, and their comparatively high frequency in this bile cfDNA study is of uncertain clinical significance, which may merit dedicated future study. *BRAF* V600E mutation was found in bile and matched tissue sample, but absent from plasma in one patient within a 10-patient resectable CCA pilot (3 iCCA, 2 distal, 5 perihilar). Though in a small pilot study, this error in detection from plasma cfDNA shows how plasma-only testing can miss a potentially actionable alteration [8].

#### 6.1.3. Intrahepatic Cholangiocarcinoma (iCCA)

iCCA is molecularly distinct from eCCA and PDAC. In the same 10-patient pilot, an *FGFR2* fusion was present in matched tissue and bile, but undetectable in plasma cfDNA. Again, the authors note that plasma-only testing would have failed to identify this patient as a candidate for targeted therapy [8]. In a broader 87-patient pancreaticobiliary cohort, druggable mutations appeared in 38% (33/87) of bile versus 18% (7/38) of plasma specimens, with bile demonstrating significantly greater sensitivity (*p* = 0.032). Overall, 23 drug-matched mutations were found across bile and plasma—5 *ERBB2*, 4 *ATM*, 3 *BRAF*, 3 *BRCA2*, 3 *NF1*, 2 *PIK3CA*, and 1 each of *BRCA1*, *IDH1*, and *PALB2* [10]. For *FGFR2* fusions, pemigatinib and futibatinib are FDA-approved for previously treated, unresectable locally advanced or metastatic iCCA.

#### 6.1.4. Gallbladder Carcinoma (GBC)

GBC differs from both iCCA and eCCA, enriched for *TP53* mutations (~47%) and ErbB pathway alterations in roughly 36.8% of cases [35]. Among the 17 studies included here, bile cfDNA data specific to gallbladder carcinoma are limited; one cohort [20] captured gallbladder cancers on extended follow-up but did not report subtype-specific mutation frequencies for GBC separately.

#### 6.1.5. Aggregate Clinical Implication Across Sites

The expanding field of precision oncology in biliary tract cancers, particularly for *FGFR2* inhibitors (pemigatinib, futibatinib), *IDH1* inhibitors (ivosidenib), and HER2-targeted agents (zanidatamab), has made genomic profiling at diagnosis a practical necessity. For patients with unresectable disease or inaccessible tissue, bile cfDNA profiling during diagnostic ERCP is a sensitive alternative to tissue biopsy. This liquid biopsy substrate effectively captures actionable genetic samples that are frequently missed by systemic blood tests. By demonstrating a significantly higher detection rate of druggable alterations compared to plasma, including the isolation of *FGFR2* fusions and *BRAF* V600E mutations that were completely absent in matched plasma circulation, bile cfDNA enables faster genotype-directed treatment planning (Table 6).

### 6.2. Whole-Genome Sequencing and Structural Complexity

WGS of bile cfDNA extends analysis beyond point mutations to the broader genomic disorder of CCA [22]. Reported findings include 53 recurrent amplification peaks and 45 deletion peaks, encompassing *EGFR*, *ERBB2*, *CDKN2A/B*, and *MYC* amplifications alongside *ARID1A* and *BRCA* pathway deletions, as well as a total of 2285 structural variants [22]. Chromothripsis, a chromosomal rearrangement event, occurred in 7 of 22 patients (31.8%), predominantly in *TP53*-mutant tumors. Homologous recombination pathway mutations were associated with markedly worse overall survival (HR 15.77; 95% CI 1.571–158.4; *p* = 0.0049), suggesting bile cfDNA WGS may serve as both a prognostic tool and a way to identify PARP inhibitor candidates [22].

## 7. Special Clinical Applications

### 7.1. PSC Surveillance and Early Cancer Detection

A compelling use of bile cfDNA is early CCA detection in PSC, where annual MRI/MRCP and CA19-9 remain inadequate. Across 63 PSC patients and 20 healthy liver-donor controls, the Bilemut assay was positive in 36.5% versus 10% (*p* = 0.0269). Among those who later developed CCA, three of four (75%) had been Bilemut-positive, with mutations preceding diagnosis by 4.9 and 6.9 months in two patients [25]. The most frequent mutations were *KRAS* (65%), *GNAS* (30%), and *TP53* (17%), with *BRAF*, *ERBB2*, *ERBB3*, and *FGFR3* each in 9% of cases. Positivity in the Bilemut assay was unrelated to cirrhosis or inflammatory bowel disease, supporting specificity for biliary mutagenesis over inflammation. Although gathered from a single pilot cohort, these findings illustrate the potential of bile cfDNA to address an unmet need in PSC surveillance and warrant prospective evaluation.

Two qualifications attach to these observations. They derive from a single pilot cohort and require independent replication before they can inform surveillance policy. The management implications of a positive result are also unsettled. No guideline addresses the mutation-positive patient with primary sclerosing cholangitis in whom imaging and cytology remain negative, and the available data do not establish what proportion of such patients will progress, over what interval, or whether earlier detection alters outcome. Reasonable interim approaches include intensified imaging surveillance and repeat sampling rather than a change in therapeutic strategy, with explicit counseling that a positive assay currently represents a research finding rather than a diagnosis. Prospective cohorts with defined surveillance intervals and adjudicated outcomes will be required before mutation status can be incorporated into surveillance algorithms.

### 7.2. Pancreaticobiliary Cancers Beyond CCA

Beyond potential early detection of CCA, bile cfDNA appears useful across pancreaticobiliary malignancies. In a cohort of 87 patients with mixed pancreaticobiliary cancers, oncogenic mutations were detected in 56% of bile versus 24% of plasma specimens (*p* = 0.005) [10]. Bile cfDNA analysis is also feasible in strictures, including PDAC and distal CCA [19], and across biliopancreatic pathology using a 28-gene panel with machine-learning classification, with bile positivity in 54% versus 17% for plasma [9].

The GRAIL Galleri test, a commercial blood-based multi-cancer early detection (MCED) assay using genome-wide targeted methylation sequencing of plasma cfDNA, reports 93.5% overall sensitivity for a pooled liver/bile duct category across all stages [36]. That figure aggregates hepatocellular carcinoma with cholangiocarcinoma and gallbladder carcinoma, and BTC-specific sensitivity has not been reported in isolation; nor has Galleri been validated in the diagnostic ERCP setting or against bile. This gap underscores the value of a bile cfDNA approach that profiles the genome and localizes cancer at the time of biliary intervention (Table 7).

### 7.3. Candidate Selection, Cost, and Turnaround Time

Translation of these data into practice requires specifying which patients should be tested and when. The available evidence supports bile cell-free DNA analysis in two groups: patients undergoing endoscopic retrograde cholangiopancreatography for an indeterminate biliary stricture in whom brush cytology is negative or inconclusive, and patients with primary sclerosing cholangitis under surveillance in whom imaging or carbohydrate antigen 19-9 raises suspicion. Routine testing at every diagnostic procedure is not supported by current data. In an unselected population, the pretest probability of malignancy is low, the consequences of a mutation detected in inflamed biliary epithelium are substantial, and no study has examined yield outside the setting of suspected malignancy.

Cost and turnaround time remain poorly characterized. No bile cell-free DNA assay is commercially available, and no study has reported per-sample cost or reporting interval for bile specifically. Published costing of a targeted 46-gene tissue panel within a national health service reported a per-patient cost of approximately 339 pounds sterling with a median turnaround of seven working days [37], which may serve as an order-of-magnitude reference for the sequencing component alone. Whether bile analysis proves more or less expensive than plasma is unresolved. Bile requires no incremental procedure where endoscopic retrograde cholangiopancreatography is already indicated, yet the long-fragment profile described in Section 3.1 may require dedicated extraction chemistry rather than the plasma-optimized kits already in routine laboratory use. Formal cost-effectiveness evaluation alongside prospective clinical validation is required.

## 8. Limitations and Implementation Challenges

Despite multiple studies demonstrating bile cfDNA’s increased mutation detection and concordance with tissue samples over plasma, bile cfDNA faces important challenges on the path to clinical translation. First, methodological heterogeneity across collection methods, extraction protocols, panel compositions (28–520 genes), and sequencing strategies makes reported performance metrics difficult to compare. Standardized reporting and protocols are lacking. Second, many published studies are single-center studies with cohort sizes of 10–96 patients and wide confidence intervals. Several of the highest sensitivities, such as 96.4% in populations with indeterminate biliary strictures [20], were obtained in populations with a high pre-test probability of malignancy and may overestimate performance in unselected cohorts. Small early studies of 10 patients [8,17] further limit generalizability. Third, specificity is a concern as *KRAS* mutations have been reported in 32.1% of non-neoplastic bile specimens [14], and overall specificity of 69.2% has been described in a stricture cohort [20], raising the possibility that mutations in non-neoplastic or chronic inflammation of the biliary epithelium may yield false-positive signals. To relieve this limitation, mutant allele frequency (MAF) thresholds, multi-gene positivity requirements, and machine-learning classification may improve specificity without sacrificing sensitivity.

Fourth, the comparative claims made throughout this review rest on studies conducted in selected populations, and the two media are not equivalently accessible. Every direct comparison of bile with plasma identified here was performed in patients already selected for biliary intervention because malignancy was suspected; performance in unselected patients, including those with indeterminate strictures of low pretest probability, remains uncharacterized. Plasma is obtained by venipuncture and may be sampled serially at negligible risk, whereas bile requires endoscopic retrograde cholangiopancreatography or percutaneous transhepatic drainage, each carrying recognized risks of pancreatitis, cholangitis, bleeding, and perforation. The analytic advantages of bile are therefore obtained without incremental procedural risk only where the procedure is independently indicated. Where it is not, the higher yield of bile must be weighed against the morbidity of obtaining it, and plasma remains the appropriate first-line medium (Table 8).

## 9. Future Research Priorities

Several priorities follow from the evidence mapped in this review.

Standardization of pre-analytical practice is the most immediate requirement. Collection volume, timing relative to contrast injection, centrifugation protocol, storage temperature, and choice of preservative tube differ across every study reviewed, and no consensus specimen-handling protocol exists. Fasting state and bile acid composition should be recorded and, where feasible, measured.

Harmonization of analytical platforms is a related priority. Panels ranging from 28 to 520 genes, droplet digital polymerase chain reaction, and whole-genome sequencing have each been applied to bile, with variant-calling thresholds set independently in each study. Comparative performance under a common reference standard has not been established, and the mutant allele frequency thresholds that distinguish malignancy from inflamed biliary epithelium require formal derivation and validation.

Prospective multicenter studies comparing bile, plasma, and tissue in the same patients, with blinded adjudication and enrollment extending to indeterminate strictures of low pretest probability, are needed to establish diagnostic performance under conditions resembling practice. Such studies should report outcomes beyond analytic detection, including the proportion of patients in whom a bile-derived result altered management.

Health economic evaluation should accompany clinical validation. The incremental cost of bile analysis relative to plasma and to repeat tissue sampling, and the threshold performance at which testing becomes cost-effective, remain unexamined.

Finally, the surveillance application in primary sclerosing cholangitis requires cohorts large enough to estimate the positive predictive value of a mutation-positive result and of sufficient duration to determine whether earlier detection improves outcome.

## 10. Conclusions

Bile cell-free DNA has emerged as a biologically enriched, analytically favorable source for studying biliary tract malignancies. Across the studies synthesized here, it yielded higher concentrations, longer fragments, and stronger mutation detection than plasma, with bile–tissue concordance exceeding plasma–tissue concordance in every direct comparison [7,11,12]. WGS of bile cfDNA revealed structural complexity, chromothripsis, and prognostically significant homologous recombination alterations [22], while targeted panels show bile captures actionable variants, including *BRAF* V600E mutations and *FGFR2* fusions that were undetectable in matched plasma [8]. Notably, preliminary evidence in PSC patients supports a role in detection of pre-clinical progression to CCA [25], which warrants further investigation given the inadequacy of current surveillance.

A greater adoption of bile cfDNA will require standardized collection and extraction protocols, approved analytical benchmarks, and prospective multicenter validation in consecutive patients with indeterminate biliary strictures. Bile-optimized extraction [18] and validated preservation conditions [21,24] are steadily lowering pre-analytical barriers. As targeted therapies for *FGFR2*-, *IDH1/2*-, and *ERBB2*-altered BTCs enter routine practice, bile cfDNA obtained during diagnostic ERCP offers a more sensitive platform to meet modern molecular profiling needs, particularly where tissue biopsy is not feasible.

## Figures and Tables

**Table 1:** Diagnostic performance of established modalities in the evaluation of indeterminate biliary strictures.

Modality	Sensitivity (%)	Specificity (%)	Principal Limitation
ERCP brush cytology	45	Not reported	Low sensitivity; yield rises with additional passes [2,4]
Fluoroscopy-guided intraductal biopsy	48	Not reported	Technically demanding; tissue often scant [2,4]
Brush cytology and intraductal biopsy combined	66	Not reported	Requires both techniques in one session [4]
Cholangioscopy with directed biopsy	74 (95% CI 62–86)	Not reported	Limited availability and added procedural time [4]
EUS-guided tissue acquisition	83 (distal strictures)	Not reported	Lower yield in perihilar disease; seeding concerns [4]
FISH on biliary brushings	58 (95% CI 49–65)	88 (95% CI 79–93)	Performance depends on the positivity criterion applied [5]
Serum CA19-9	72 (95% CI 70–75)	84 (95% CI 82–85)	Elevated in obstruction, cholangitis, and PSC; cutoffs vary [6]

CI = confidence interval; ERCP = endoscopic retrograde cholangiopancreatography; EUS = endoscopic ultrasound; FISH = fluorescence in situ hybridization; CA19-9 = carbohydrate antigen 19-9; PSC = primary sclerosing cholangitis. Values are pooled estimates from the cited systematic reviews and guideline syntheses; specificity was not reported for all modalities.

**Table 2:** Characteristics of included studies.

First Author (Year)	Journal	*n*	Cancer Type	Analytical Platform	Key Finding
Shi (2009) [14]	J Mol Diagn	44 bile, 20 serum	Neoplastic biliary lesions	LigAmp (*KRAS*)	*KRAS* in 87.5% of neoplastic bile vs. 32.1% of benign; 2% MAF threshold improves specificity
Shen (2019) [17]	Oncol Rep	10	GBC (*n* = 4), CCA (*n* = 6)	Panel NGS + gel electrophoresis	Bile cfDNA predominantly ~6000 bp; SNV sensitivity 94.7%, CNV sensitivity 75.0%
Driescher (2020) [7]	Cancers	Mixed cohort	CCA, PDAC, controls	Panel NGS (ERCP bile)	Bile detected 96.2% of tissue mutations vs. 31.6% in plasma; specificity 100%
Han (2021) [11]	Cancers	42	CCA, GBC, ampullary	ddPCR (*KRAS*)	80% bile–tissue concordance vs. 42.9% plasma; *KRAS*+ associated with worse OS (*p* = 0.018)
Gou (2021) [13]	ESMO Open	28	CCA, GBC, PDAC	Panel NGS	71.4% bile vs. 53.6% plasma detection; CA19-9 + bile cfDNA = 96.4% sensitivity
Li (2022) [12]	Front Oncol	13	CCA, GBC	Panel NGS	Bile cfDNA 1918 vs. 63.1 ng/mL plasma (*p* = 0.0017); tissue consistency 90% vs. 35%
Shen (2022) [18]	Front Oncol	N/A (methodology)	N/A	3D-BCF vs. 4 commercial kits	3D-BCF highest yield for 40 bp–10 kb fragments across all size ranges
Nagai (2022) [19]	Diagnostics	50	CCA (*n* = 32), PDAC, benign	50-gene NGS panel	Sensitivity 53% vs. cytology 19% (*p* = 0.016); fragments 8000–10,000 bp
Arechederra (2022) [20]	Gut	68	Biliary strictures	Bilemut (52-gene Oncomine)	Sensitivity 96.4%, specificity 69.2%; 54% actionable mutations identified
Ohyama (2023) [10]	Cancer	87	CCA, PDAC, ampullary	60-gene NGS panel	56% bile vs. 24% plasma (*p* = 0.005); druggable mutations 38% vs. 18%
Yang (2024) [21]	Transl Cancer Res	N/A (pre-analytical)	N/A	Stability testing	Stable 10 days at RT in preservative tubes; −20 °C required for standard tubes
Yin (2024) [22]	Genomics	40 CCA	iCCA, eCCA, pCCA	WGS	Chromothripsis in 7/22; HR pathway mutations: HR 15.77 for worse OS (*p* = 0.0049)
Ito (2024) [9]	Cancer Sci	24	CCA, PDAC, ampullary	LiquidPlex 28-gene + ML	54% bile vs. 17% plasma positive; 68.2× enrichment vs. plasma
Liu (2025) [8]	iLIVER	10	Resectable CCA	520-gene NGS panel	Bile 70% vs. plasma 10%; *BRAF*/*FGFR2* detected in bile but not plasma
He (2025) [23]	J Gastrointest Cancer	96	CCA (*n* = 48) vs. gallstones	Bile cfDNA (Syncytin-1, *SLC7A11*)	Syncytin-1 + *SLC7A11* + CA19-9 AUC 0.927 vs. CA19-9 alone 0.857
Han (2025) [24]	Sci Rep	N/A (pre-analytical)	N/A	Stability testing	Proteins and ctDNA stable at RT for up to 7 h
Arechederra (2025) [25]	Liver Int	63 PSC + 20 controls	PSC surveillance	Bilemut (52-gene Oncomine)	36.5% PSC Bilemut-positive; early CCA detection 4.9–6.9 months pre-diagnosis

CCA = cholangiocarcinoma; GBC = gallbladder carcinoma; PDAC = pancreatic ductal adenocarcinoma; PSC = primary sclerosing cholangitis; NGS = next-generation sequencing; ddPCR = droplet digital PCR; WGS = whole-genome sequencing; ML = machine learning; RT = room temperature; OS = overall survival; HR = hazard ratio; MAF = mutant allele frequency; SNV = single-nucleotide variant; CNV = copy number variant; CA19-9 = carbohydrate antigen 19-9; N/A = not applicable.

**Table 3:** Pre-analytical considerations for bile cell-free DNA.

Study	Collection Method	Extraction Platform	Stability Conditions	Key Pre-Analytical Finding
Shen et al. (2019) [17]	PTCD vs. intraoperative	Gel electrophoresis + panel NGS	Standard freezing	No significant difference between PTCD and intraoperative collection in cfDNA quantity or SNV detection
Shen et al. (2022) [18]	N/A	3D-BCF (novel) vs. 4 commercial kits	N/A	3D-BCF achieved the highest cfDNA yield across 40 bp–10 kb; commercial kits miss long fragments
Yang et al. (2024) [21]	N/A	Multiple kits tested	RT up to 10 days (preservative); −20 °C (standard)	Preservative tubes extend RT stability to 10 days; standard tubes require −20 °C
Han et al. (2025) [24]	Drainage bile	N/A	RT stable up to 7 h	ctDNA and proteins stable at RT for 7 h; compatible with standard ERCP workflow

PTCD = percutaneous transhepatic cholangiodrainage; ERCP = endoscopic retrograde cholangiopancreatography; RT = room temperature; SNV = single-nucleotide variant; N/A = not applicable.

**Table 4:** Diagnostic performance of bile cfDNA across studies.

Study	*n*	Detection/Sensitivity	Specificity	Plasma Comparison	Additional Findings
Shi (2009) [14]	44 bile	87.5% neoplastic bile	67.9% †	Serum: 81.8%	2% MAF threshold: 57% cancer vs. 7% benign (*p* < 0.01)
Shen (2019) [17]	10	SNV 94.7%; CNV 75.0%	SNV 99.9%; CNV 98.9%	Not reported	No difference between PTCD and intraoperative collection
Han (2021) [11]	42	80% bile–tissue concordance	N/A	Plasma–tissue: 42.9%	*KRAS* MAF predicts OS (*p* = 0.018)
Driescher (2020) [7]	Mixed cohort	96.2% mutation detection	100%	Plasma: 31.6%	Bile outperformed plasma across all mutation categories
Gou (2021) [13]	28	71.4%	Not reported	Plasma: 53.6%	CA19-9 + bile cfDNA: 96.4% sensitivity
Li (2022) [12]	13	90% tissue consistency	Not reported	Plasma: 35%	Bile cfDNA 1918 vs. 63.1 ng/mL (*p* = 0.0017)
Nagai (2022) [19]	50	53% (malignancy detection)	75%; PPV 89.5%	Not reported	Brush cytology sensitivity only 19% (*p* = 0.016)
Arechederra (2022) [20]	68	96.4%	69.2%	Not directly compared	54% actionable mutations; 100% sensitivity for indeterminate strictures
Ohyama (2023) [10]	87	56%	Not reported	Plasma: 24% (*p* = 0.005)	Druggable mutations: 38% bile vs. 18% plasma
Ito (2024) [9]	24	54% (13/24)	Not reported	Plasma: 17% (4/17)	68.2× cfDNA enrichment vs. plasma
Liu (2025) [8]	10	70%	PPV 90.2%	Plasma: 10%	*BRAF*/*FGFR2* in bile + tissue, not plasma; TMB comparable to tissue

† Specificity estimated from 28 non-neoplastic samples at 2% MAF threshold. MAF = mutant allele frequency; PPV = positive predictive value; OS = overall survival; TMB = tumor mutation burden; SNV = single-nucleotide variant; CNV = copy number variant; CA19-9 = carbohydrate antigen 19-9; N/A = not applicable.

**Table 5:** Mutational spectrum detected in bile cell-free DNA across studies.

Study	*n*	Most Frequent Mutations	Actionable Alterations	Prognostic/Clinical Relevance	Anatomic Site of Collection	Type of Cancer
Shi (2009) [14]	44 bile	*KRAS* (87.5% neoplastic)	*KRAS*	2% MAF threshold separates malignant from benign (*p* < 0.01)	Bile duct (biliary stricture specimens)	Biliary Tract Cancer
Shen (2019) [17]	10	SNV/Indel panel; CNV detected	CNV	CNV sensitivity reduced in stage II and distal bile duct tumors	Bile (method of collection not specified)	Biliary Tract Cancer
Arechederra (2022) [20]	68	*KRAS* (71.9%), *TP53* (47.4%), *ERBB3* (22.8%), *GNAS* (15.8%), *ERBB2* (8.8%)	*ERBB2*, *FGFR2*, *IDH1/2*	54% overall actionable; 100% sensitivity for indeterminate strictures	Bile collected at ERCP from biliary strictures: Intrahepatic: 3 (4.4%) Perihilar: 17 (25%) Distal: 48 (70.6%)	CCA (*n* = 24) PDAC (*n* = 9)
Ohyama (2023) [10]	87	60-gene profiling; bile identified more alterations than plasma	38% druggable in bile vs. 18% plasma	23 drug-matched mutations identified	Bile supernatant + precipitate collected during biliary drainage procedures (ERCP or PTCD)	CCA *n* = 59 (hilar 32, distal 21, iCCA *n* = 6 *) GBC *n* = 12 Pancreatic cancer *n* = 16
Yin (2024) [22]	40 *	*TP53* 27.3%, *KRAS* 9.1%; RTK/RAS pathway in 8 patients	*ERBB2*, *EGFR* amplifications; *BRCA* deletions	Chromothripsis in 31.8%; HR mutations HR 15.77 (*p* = 0.0049)	Bile collected via biliary drainage (ERCP or PTBD)	Cholangiocarcinoma
Liu (2025) [8]	10	*BRAF* V600E, *FGFR2* fusion	*BRAF* V600E, *FGFR2*	*BRAF*/*FGFR2* in bile + tissue but not plasma; TMB bile ≈ tissue	Bile collected at surgery (resectable cases): iCCA *n* = 3 Perihilar CCA *n* = 5 Distal CCA *n* = 2 No GBC or PDAC included [8]	iCCA *n* = 3 Perihilar CCA *n* = 5 Distal CCA *n* = 2
Arechederra (2025) [25]	63 PSC	*KRAS* 65%, *GNAS* 30%, *TP53* 17%, *BRAF*/*ERBB2*/*ERBB3*/*FGFR3* 9%	*ERBB2*, *FGFR3*, *BRAF*	CCA detected 4.9–6.9 months before clinical diagnosis	Bile collected via ERCP in PSC patients under surveillance	PSC patients (surveillance cohort) CCA detected on follow-up
He (2025) [23]	96	Syncytin-1, *SLC7A11* overexpressed	Novel bile biomarkers	Syncytin-1 + *SLC7A11* + CA19-9 AUC 0.927; Syncytin-1 AUC 0.805, specificity 87.0%	Bile (collection method not specified as ERCP vs. drainage)	Cholangiocarcinoma

* 22 patients had adequate cell-free DNA quality for whole-genome sequencing analysis. AUC = area under the receiver operating characteristic curve; CA19-9 = carbohydrate antigen 19-9; CCA = cholangiocarcinoma; cfDNA = cell-free DNA; CNV = copy number variant; ERCP = endoscopic retrograde cholangiopancreatography; GBC = gallbladder carcinoma; HR = hazard ratio; iCCA = intrahepatic cholangiocarcinoma; MAF = mutant allele frequency; OS = overall survival; PDAC = pancreatic ductal adenocarcinoma; PSC = primary sclerosing cholangitis; PTBD = percutaneous transhepatic biliary drainage; PTCD = percutaneous transhepatic cholangiodrainage; RTK = receptor tyrosine kinase; SNV = single-nucleotide variant; TMB = tumor mutation burden; WGS = whole-genome sequencing.

**Table 6:** Actionable alterations detected in bile cell-free DNA and their therapeutic relevance.

Alteration	Predominant Site	Reported Frequency in Bile cfDNA	Matched Agent	Status
*KRAS*	PDAC, eCCA	22% (7/32) of pancreaticobiliary bile specimens [19]	G12C inhibitors	Not approved in BTC; G12C < 2% of PDAC alleles [30,31]
*TP53*	All sites	47.4% of Bilemut-positive cases [20]; 27.3% by WGS [22]	None	Prognostic only
*GNAS*	IPMN-associated PDAC	15.8–30% [20,25]	None	May stratify IPMN progression
*ERBB2* amplification	eCCA, GBC	8.8% [20]	Zanidatamab	FDA accelerated approval, November 2024 [34]
*ERBB3*	eCCA	22.8% [20]	None	Clinical significance uncertain
*BRAF* V600E	CCA	Detected in bile and matched tissue, absent in plasma [8]	*BRAF*-directed therapy	Investigational in BTC
*FGFR2* fusion	iCCA	Detected in bile, undetectable in paired plasma [8]	Pemigatinib, futibatinib	FDA approved for previously treated iCCA
*IDH1*	iCCA	1 of 23 drug-matched mutations [10]	Ivosidenib	FDA approved
Homologous recombination pathway	CCA	Associated with worse overall survival (HR 15.77) [22]	PARP inhibitors	Investigational

BTC = biliary tract cancer; CCA = cholangiocarcinoma; cfDNA = cell-free DNA; eCCA = extrahepatic cholangiocarcinoma; FDA = United States Food and Drug Administration; GBC = gallbladder carcinoma; HR = hazard ratio; iCCA = intrahepatic cholangiocarcinoma; IPMN = intraductal papillary mucinous neoplasm; PARP = poly(ADP-ribose) polymerase; PDAC = pancreatic ductal adenocarcinoma; WGS = whole-genome sequencing. Frequencies reflect detection in bile rather than underlying tumor genotype.

**Table 7:** Reported and proposed clinical applications of bile cell-free DNA.

Application	Supporting Evidence	Current Status
Diagnosis of indeterminate biliary stricture	Sensitivity 96.4%, specificity 69.2% in 68 stricture patients [20]	Investigational
Complement to brush cytology	Sensitivity 53% versus 19% for cytology in 50 patients [19]	Investigational; roles appear complementary
Genotype-directed therapy selection	Druggable mutations in 38% of bile versus 18% of plasma specimens [10]	Investigational; highest reported yield
Combination with serum CA19-9	Sensitivity 96.4% combined versus 71.4% for bile alone [13]	Investigational
Early detection during PSC surveillance	36.5% positive versus 10% of controls; three of four subsequent CCA cases positive before diagnosis [25]	Single pilot cohort; requires replication
Prognostic stratification	*KRAS* mutant allele frequency associated with worse survival [11]; homologous recombination mutations HR 15.77 [22]	Exploratory
Detection across pancreaticobiliary malignancy	Mutations in 56% of bile versus 24% of plasma specimens [10]; 54% versus 17% in a second cohort [9]	Investigational

CA19-9 = carbohydrate antigen 19-9; CCA = cholangiocarcinoma; HR = hazard ratio; PSC = primary sclerosing cholangitis. No application listed has been validated prospectively or shown to alter clinical management.

**Table 8:** Strengths and limitations of bile cell-free DNA as a liquid biopsy medium.

Domain	Strength	Limitation
Yield	Concentrations exceed paired plasma by one to two orders of magnitude [12,20], with 68.2-fold enrichment reported [9]	No commercially available assay; no standardized extraction protocol [18]
Fragment profile	Long fragments of 8000–10,000 bp permit structural and copy-number analysis [17,19,22]	Plasma-optimized extraction kits underperform on long fragments [18]
Tissue concordance	Exceeded plasma in every direct comparison reported [7,11,12]	Several estimates derive from fewer than 15 paired specimens [8,12]
Actionability	Captures *BRAF* V600E and *FGFR2* fusions absent from paired plasma [8]	No prospective study has demonstrated altered management or outcome
Access	Obtained during ERCP or percutaneous drainage at no incremental procedural risk where already indicated	Requires an invasive procedure; unsuitable for serial or screening use
Specificity	Mutant allele frequency thresholds and machine-learning classification may improve discrimination [9]	*KRAS* detected in 32.1% of non-neoplastic bile [14]; specificity 69.2% in one stricture cohort [20]
Pre-analytical stability	Preservative tubes maintain integrity for up to 10 days at room temperature [21]	Standard blood collection tubes unvalidated in bile; bile acid composition uncontrolled [26]
Evidence base	Seventeen primary studies spanning biology, platforms, and diagnostic performance	Predominantly single-center and retrospective, 10–96 patients, without blinded adjudication

bp = base pairs; ERCP = endoscopic retrograde cholangiopancreatography.

## Data Availability

No new data were created or analyzed in this study. All data discussed are available in the cited published articles.

## Supplementary Materials

The following supporting information can be downloaded at: https://www.mdpi.com/article/10.3390/cancers18203251/s1. Table S1. Embase search strategy and yield; Table S2. PubMed/MEDLINE search strategy and yield; Table S3. Derivation of each value in Figure 1.
