# Supplementary material for: Bile Cell-Free DNA as a Liquid Biopsy for Biliary Tract Malignancies: A Scoping Review
Source: Cancers (Basel). 2026 Oct 9;18(20):3251. doi: 10.3390/cancers18203251 (PMC13702538; doi:10.3390/cancers18203251)
Supplement: Supplementary file 1 [file cancers-18-03251-s001.zip › cancers-4594199-supplementary.pdf]

Supplementary Material

Bile Cell-Free DNA as a Liquid Biopsy for Biliary Tract Malignancies: A Scoping Review

Supplementary Table S1. Embase search strategy and yield

Embase (Embase.com), searched 14 September 2026, no date or language restrictions.

#	Search string	Records
1	'cell-free dna' AND 'biliary'	195
2	'circulating tumor dna' AND 'cholangiocarcinoma'	261
3	'bile cell-free dna' AND 'cholangiocarcinoma'	6
4	'bile cell-free dna' AND 'pancreatic cancer'	3
5	'bile cell-free dna' AND 'plasma'	7
6	'bile cell-free dna' AND 'circulating tumor dna'	1
7	'bile cell-free dna' AND 'liquid biopsy'	11
8	'plasma circulating dna' AND 'liquid biopsy'	9
9	'bile' AND 'cell-free dna'	202
10	'bile' AND 'liquid biopsy'	510
11	'bile' AND 'circulating tumor dna' AND 'plasma'	95
**12**	**#1 OR #2 OR #3 OR #4 OR #5 OR #6 OR #7 OR #8 OR #9 OR #10 OR #11 (deduplicated union)**	**939**

Lines 1–11 sum to 1,300; line 12 removes 361 overlapping records (27.8%). Only line 12 contributes to Figure 1.

Supplementary Table S2. PubMed/MEDLINE search strategy and yield

PubMed/MEDLINE, searched 14 September 2026, no date or language restrictions.

#	Search string	Records
1	bile AND liquid biopsy	200
2	bile AND cell-free DNA	285
3	circulating tumor DNA AND cholangiocarcinoma	135
4	bile cell-free DNA AND circulating tumor DNA	53
5	bile cell-free DNA AND cholangiocarcinoma	96
6	bile cell-free DNA AND pancreatic cancer	34
7	bile cell-free DNA AND plasma	51
8	bile cell-free DNA AND liquid biopsy	42
9	plasma circulating tumor DNA AND liquid biopsy	1,689
10	(plasma circulating tumor DNA) AND (liquid biopsy) AND (bile cell-free DNA)	14
11	(plasma circulating tumor DNA) AND (cholangiocarcinoma)	31
**12**	**#1 OR #2 OR #3 OR #4 OR #5 OR #6 OR #7 OR #8 OR #9 OR #10 OR #11 (deduplicated union)**	**2,201**

Lines 1–11 sum to 2,630; line 12 removes 429 overlapping records (16.3%). Only line 12 contributes to Figure 1. Line 9 retrieves predominantly plasma-based studies and contributes up to 1,689 of the 2,201 records; plasma cell-free DNA terms were searched deliberately so that plasma-only studies could be identified as the comparator and excluded at screening.

Supplementary Table S3. Derivation of each value in Figure 1

Step	n	Derivation
PubMed/MEDLINE union (Table S2, line 12)	2,201	
Embase union (Table S1, line 12)	939	
Total records identified	3,140	2,201 + 939
Duplicate records removed (Zotero, manual)	− 587	18.7% of 3,140
Records screened	2,553	3,140 − 587
Excluded at title/abstract	− 2,520	2,553 − 33

Reports sought for retrieval	33	
Reports not retrieved	− 0	
Reports assessed for eligibility	33	
Excluded at full text	− 17	8 plasma/serum only + 9 abstract only
Studies included from databases	16	33 − 17
Studies included from citation searching	+ 1	
**Total studies included in review**	**17**	**16 + 1**

Duplicate removal was performed manually in Zotero. Title and abstract screening and full-text assessment were carried out independently by two reviewers, with disagreements resolved by discussion.
